# Clinically high-risk breast cancer displays markedly discordant molecular risk predictions between the MammaPrint and EndoPredict tests

**DOI:** 10.1038/s41416-020-0838-2

**Published:** 2020-04-27

**Authors:** Stephan Wenzel Jahn, Andreas Bösl, Oleksiy Tsybrovskyy, Christine Gruber-Rossipal, Ruth Helfgott, Florian Fitzal, Michael Knauer, Marija Balic, Zerina Jasarevic, Felix Offner, Farid Moinfar

**Affiliations:** 1 Diagnostic and Research Institute of Pathology, Medical University of Graz, Neue Stiftingtalstraße 6, 8010 Graz, Austria; 2 Institute of Pathology, Schwerpunktkrankenhaus Feldkirch, Carinagasse 47, 6800 Feldkirch, Austria; 3 Department of Pathology, Ordensklinikum/Hospital of the Sisters of Charity, Seilerstätte 4, 4010 Linz, Austria; 4 Vincent Academy of Pathology, Seilerstätte 4, 4010 Linz, Austria; 5 Department of Surgery, Ordensklinikum/Hospital of the Sisters of Charity, Seilerstätte 4, 4010 Linz, Austria; 6 Department of Surgery, Comprehensive Cancer Center, Medical University of Vienna, Währinger Gürtel 18-20, 1090 Vienna, Austria; 7 Breast Center Eastern Switzerland, Schuppisstrasse 10, 9016 St. Gallen, Switzerland; 8 Division of Oncology, Department of Internal Medicine, Clinical Department of Oncology, Medical University of Graz, Auenbruggerplatz 15, 8036 Graz, Austria

**Keywords:** Breast cancer, Prognostic markers

## Abstract

Inter-test concordance between the MammaPrint and the EndoPredict tests used to predict the risk of recurrence in breast cancer was evaluated in 94 oestrogen receptor-positive, HER2-negative breast cancers. We correlated histopathological data with clinical risk estimation as defined in the MINDACT trial. 42.6% (40/94) of cases were high-risk by MammaPrint, 44.7% (42/94) by EndoPredict (EPclin), and 45.7% (43/94) by clinical risk definition. Thirty-six percent of genomic risk predictions were discordant with a low inter-test correlation between EndoPredict and MammaPrint (*p* = 0.012; κ = 0.27, 95% CI [0.069, 0.46]). Clinical risk stratification did not correlate with MammaPrint (*p* = 0.476) but highly correlated with EndoPredict (*p* < 0.001). Consequently, clinically high-risk tumours (*n* = 43) were more frequently high-risk by EndoPredict than by MammaPrint (76.6% vs. 46.5%, *p* = 0.004), with 44% of cases discordantly classified and no significant association between genomic risk predictions (*p* = 0.294). Clinicians need to be aware that clinical pre-stratification can profoundly influence multigenomic test performance.

## Introduction

Up to 40% of women with early breast cancer receive adjuvant chemotherapy at the price of considerable overtreatment as 70–80% percent of patients are estimated to equally have survived without it.^1^ Multigenomic assays can aid in the deliberation on adjuvant chemotherapy. The selection of proprietary tests is often highly individual, depending on reimbursement, geographic region and personal preferences of the oncologist. We directly compared two commonly used tests, namely MammaPrint® (MP) (Agendia, Amsterdam, Netherlands, EU), and EndoPredict® (EP) (Myriad International, Cologne, Germany, EU) as to our knowledge only two smaller studies have so far addressed the concordance between these two tests.^2,3^ After parallel analyses from identical tumours, we correlated risk-stratifications with clinical and histological data. To apply both assays within their designated specifications, we evaluated the tests on a cohort of ER+, HER2−, TNM Stage I and II breast cancers below 5 cm in diameter with up to three positive lymph nodes.

## Material and methods

Fifty-six cases from the Department of Pathology, Hospital of the Sisters of Charity, Linz Austria (ethics committee approval 31/09, Ordensklinikum/Hospital of the Sisters of Charity, Linz Austria) were analysed in the course of this study. MP/EP risk-prediction data from another 38 cases previously published by Bösl et al.,^2^ selected for compliant inclusion criteria were evaluated after the authors were contacted (Schwerpunktrankenhaus Feldkirch Austria). Tumours from Linz had received MP testing on routine clinical requests between 2010 and 2016 and were retested with EP for study purposes. For clinical data of the combined cohort (*n* = 94) see Supplemental Table 1.

MammaPrint testing was performed centrally by Agendia, Europe. EndoPredict testing was performed at the Department of Pathology, Ordensklinikum/Hospital of the Sisters of Charity, Austria according to manufacturer´s instructions. We computed EPscore and through the addition of pT and pN information, the EPclin. We used categorical “low”/“high” risk classifications for MP, EPscore, and EPclin for statistical analyses. For better readability, mention of “EP” denotes final EPclin results. The methodology for the external cohort is described in Bösl et al.,^2^ and proliferative activity/histological grade was evaluated according to the criteria detailed below. ER/PR positivity was defined as ≥1% positive cells by immunohistochemistry. Risk stratifications were correlated with histological grade 1–3, three-tiered Ki-67 stratification rounded to the nearest 5% (<10%/10–30%/>30%), progesterone receptor positivity, pathological T-stage (applicable: pT1b/T1c/T2) and nodal status (pN0/N1). We performed clinical risk assessment as described in the MINDACT trial^4^ (Supplemental appendix Table S13): For ER+/PR+, HER-2-negative tumours pathological T and N stage, as well as grade, were used for classification into “C-low” or “C-high” risk groups.

We used SPSS, V.23 (IBM, USA) and R-scripts^5^ for statistical analyses: Two-sided Chi-square test or Fisher´s exact test for associations between categorical variables, Cohen´s Kappa and Spearman´s Rho to quantify correlations, McNemar tests to compare risk-prediction frequencies. Significance was defined as *p* ≤ 0.05 with all Spearman´s Rho *p* ≤ 0.05 if not further specified.

## Results

**Fig. 1:**
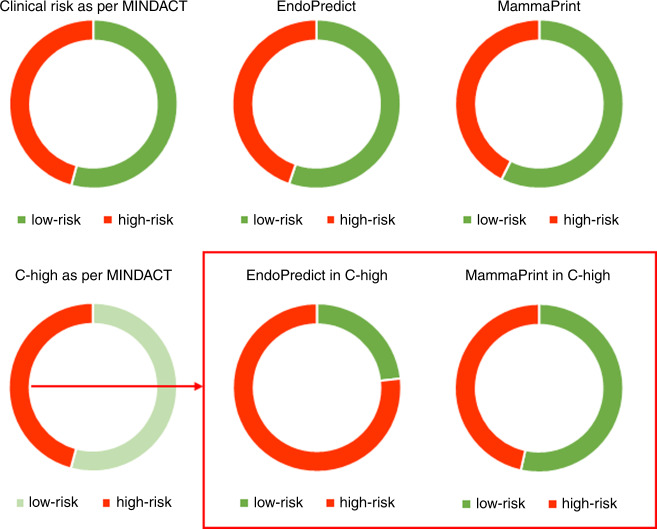
Results as proportions of low and high-risk predictions. Top row displays whole cohort (*n* = 94) Left: Stratified according to clinical criteria of the MINDACT study (Cardoso et al., NEJM 2016), middle: with EndoPredict, right: with MammaPrint. Bottom row displays clinically high-risk cases. Left: Clinically high-risk cases (*n* = 43) further stratified with EndoPredict (middle) and MammaPrint (right).

Ninety-four cases were amenable to evaluation comprising the 56 cases with de-novo molecular testing and the external data set. 79.8% of cases were high-risk by EPscore, 44.7% by EPclin, and 42.6% by MP. Histopathological Ki-67 index was significantly associated with EPclin and MP, while pT, pN and clinical risk as per MINDACT correlated with EPclin but not MP. For multigenomic/clinical correlation, see Supplemental Table 2, for crosstabulation of test results and risk-predictions per case, see Supplemental Tables 3 and 4. Case per case MP to EPclin risk predictions were discordant in 36%. MP to EPclin risk was significantly associated (*p* = 0.01). Measure of agreement was κ = 0.27, 95% CI [0.069, 0.46] and 99% CI [0.0075, 0.52]. In clinically high-risk cases (*n* = 43), genomic high-risk predictions were 93% by EPscore, 76.7% by EPclin, and 46.5% by MP. Discordant risk predictions now increased to 44% and MP to EPclin results failed to show a significant association (*p* = 0.294, κ = 0.15, 95% CI[−0.089, 0.39]). Clinically high-risk cases were 65% more frequently high-risk by EPclin than by MP (*p* = 0.004). Figure 1 displays the results for clinical and multigenomic risk stratification.

## Discussion

To our knowledge, our study is the most comprehensive EP to MP comparison to date and the first to look at inter-test performance in clinically high-risk tumours. Evaluation of 94 ER+/HER2− breast cancers demonstrated an almost equal rate of high-risk predictions for the whole cohort (42.6% vs. 44.7%) but with contradictory predictions for the same tumour in more than a third of cases (36%). This MP to EP discordance rate is in agreement with Bösl et al.^2^ at 34%, Pelaez-Garcia et al.^3^ at 27.5%, and at 32% approximated in silico from microarray data.^6^ For clinically applied diagnostics a measure of agreement of κ = 0.27 is disappointing. The upper limit of the 99% CI at κ = 0.52 implies that inter-test agreement must be expected to remain unsatisfactory even in larger cohorts. The molecular signatures (MP and EPscore) are so different that no statistical association in close to a hundred cases was discernible. Only after the addition of clinical information (EPclin), a significant inter-test association was seen. Therefore clinical information in only one of the tests (EP) is not the underlying cause of discordance as previously hypothesised^3^ but on the contrary increases concordance. Assuming an actual 20–30% risk of recurrence,^1^ both tests substantially overestimate recurrence, albeit partially in different patients.

Also, we investigated both tests in clinically high-risk cases. A recent ASCO practice guideline update^7^ for MP advises to only perform testing in tumours with high clinical risk according to the criteria of the MINDACT trial.^4^ The study combined clinical and genomic risk-prediction. Patients with clinically low-risk tumours received no benefit from chemotherapy irrespective of genomic risk, thus rendering molecular testing unnecessary. Furthermore, only patients at high clinical and high genomic risk were unequivocally advised to receive adjuvant chemotherapy. In clinically high-risk tumours differences between MP and EP increased so that a high-risk report was now 65% more likely by EP than by MP (76.7% vs. 46.5%), and this difference was statistically significant (*p* = 0.004). Almost every other case (44%) was now discordantly classified. The clinical risk did not correlate with MP (*p* = 0.481) but weakly correlated with EPscore (Rho = 0.303). The correlation increased after clinical information (pT, pN) was used to derive EPclin (Rho = 0.592). EPclin, as well as clinical risk stratification as per MINDACT both draw on pT/pN information^4,8^ as the two most important clinical prognostic predictors. Consequently, clinical information is redundantly evaluated by EPclin in clinically high-risk tumours. The point is illustrated by a reduction of high-risk predictions from 80% (EPscore) to 45% (EPclin) for the whole cohort, compared to only a minor reduction from 93% (EPscore) to 76% (EPclin) in clinically high-risk cases. Our data compare well to the 77% genomic high-risk predictions by EPclin in the node-positive subgroup of the recent study by Sestak et al.^9^ and the 39% of clinically high-risk (HR+/HER2−) cases by MP in the MINDACT trial.^4^ Clinicians need to be aware that clinical pre-stratification can significantly impact multigenomic test performance. As the first prospective trial on EP risk-prediction is ongoing at four years follow-up,^10^ our results confirm the need for further comparative prospective clinical trials, with a particular focus on test performance in clinically high-risk tumours.

## Supplementary information

Supplemental table 1

Supplemental table 2

Supplemental table 3

Supplemental table 4

## Data Availability

Data used for analysis are listed in Supplemental Table 4.
